# The Micro-Shear bond strength of different cements to commercially pure titanium

**DOI:** 10.4317/jced.56022

**Published:** 2019-09-01

**Authors:** Mohammadreza Nakhaei, Zeinab Fendereski, Samin Alavi, Hamideh-Sadat Mohammadipour

**Affiliations:** 1 Associate Professor of Prosthodontics, Dental Materials Research Center, Mashhad University of Medical Sciences, Mashhad, Iran. Department of Prosthodontics, School of Dentistry, Mashhad University of Medical Sciences, Mashhad, Iran; 2 Dental Student, School of Dentistry, Mashhad University of Medical Sciences, Mashhad, Iran; 3 Assistant Professor of Restorative Dentistry, Department of Cosmetic and Restorative Dentistry, School of Dentistry, Mashhad University of Medical Sciences, Mashhad, Iran; 4 Assistant Professor of Restorative Dentistry, Dental Research Center, Mashhad University of Medical Sciences, Mashhad, Iran. Department of Cosmetic and Restorative Dentistry, School of Dentistry, Mashhad University of Medical Sciences, Mashhad, Iran

## Abstract

**Background:**

The most appropriate luting agent for attaching the prefabricated Ti-based insert of hybrid abutments to its ceramic component has not yet been determined. This study was done aimed at examining the micro-shear bond strength (μSBS) of different cements to commercially pure titanium (Cp Ti).

**Material and Methods:**

A total of 100 milled cubes of Cp Ti was airborne-particle abraded using 250 μm aluminum oxide particles. Specimens were then divided into 5 groups (n=20) according to the type of resin cement used: (1) Panavia F.2, (2) Rely X U200, (3) Panavia SA LUTING Plus, (4) GC Fuji I, and (5) GC FujiCEM 2. After 24h storage, half of the samples were subjected to 5000 cycles of thermal aging. Next, the bonded samples were tested in the micro-shear mode. Data (MPa) were analyzed using a two-way ANOVA and the post hoc Tukey test (α=0.05). After debonding, each sample was examined for the failure mode classification.

**Results:**

The highest μSBS value in the study cements was obtained for Panavia F.2 cement (*P*<0.001) with no significant difference with Rely X U200 (*P*=0.07). The μSBS values of both GI-based cements were significantly lower than those of resin cements. Thermal aging decreased the μSBS values of all groups (*P*=0.003) significantly. The mainly occurred failure mode in all groups was the adhesive feature.

**Conclusions:**

Resin cements demonstrated acceptable bonding to Cp Ti, yet Gl-based cements did not. From among the cements examined, Panavia F.2 can be considered as the best option for bonding to Ti.

** Key words:**Bond strength, Glass ionomer, Hybride abutment, Resin cement, Titanium.

## Introduction

Titanium (Ti) with some features like favorable Bioco patibility, corrosive resistance, and sufficient mechanical strength is the preferred option for use in dental implant abutments (1).

However, the alloy’s gray metallic color poses an esthtic challenge, especially in submucosal peri-implant tissues (2).

In spite of favorable esthetically outcomes of the Zirconia abutment, several drawbacks, including the fracture at the abutment’s neck and the wear at the implant connection have limited its application. In order to avoid such problems and produce more natural esthetic developed, the bi-component hybrid abutment has been dev loped. The hybrid abutment is composed of two components, including a prefabricated Ti-based insert (the Ti-base abutment) and a zirconia or lithium disilicate ceramic component. Normally, the ceramic component gets attached to the Ti-based abutment through the cementation process (3-7).

Thus, the hybrid abutment is developed by combining the Ti strength and the esthetic nature of ceramic materials (8). Drawing upon previous statements, the clinical success of the hybrid abutment depends on the cementation technique used to create a link between the ceramic and the metal (Ti).

Various categories of luting cements, including conventional and resin cements have been proposed for prosthetic cementation. Due to the lack of adhesion to dental tissues, conventional cements, such as zink phosphate and polycarboxylate were substituted by glass ionomer (Gl), resin modified glass ionomer (RMGl), and later by resin cements. Some features, such as high the ability to adhere to the tooth structure as well as metal and ceramic

substrates, a wide range of esthetic shades, favorable mechanical properties, a high strength, an excellent retention property, and poor solubility in oral environments (9,10) turn resin cements into the first option in the cementation of indirect restorations. According to the clinical steps required to prepare the substrate prior to cementation, the resin cements were categorized in three sub-groups, including etch-and-rinse, self-etch, and self-adhesive luting agents. The clinical application of the etch-and-rinse and self-etch cements could be more technique sensitive, time-consuming, and prone to handling errors. The self-adhesive resin cements were created to overcome some of the shortcomings of both conventional and resin cements, using a single component. The simultaneous etching and bonding of the substrate could reduce the technique sensitivity of the former resin cements. Besides, the presence of multifunctional monomers, such as 10-MDP in the resin matrix of these cements makes them more suitable for the cementation of various restorative materials, including the composite resin, ceramic-based and metal-based materials, as well as the tooth structure (11).

The current literature provides limited information on the bond strength (BS) of different cements to Ti-based alloy abutments in hybrid abutment systems. Most of the previous studies have investigated the effects of different surface treatment methods on the BS of resin cements to Ti (3-6,12) or have otherwise focused on Ti posts cemented into the canal space (13-16).

Materials used in the oral cavity are subject to the effects of humidity and temperature variations that can influence the durability of the resin bonding to metal surfaces (7).

Therefore, it is crucial to create strong and durable adhesion between Ti-based abutments and ceramics using a luting agent to withstand thermal and mechanical changes in the oral cavity.

Therefore, the current study was done aimed at evaluating the micro-shear bond strength (μSBS) of several types of cements including, the self-etch resin cement (Panavia F.2), the self-adhesive cement (Rely X U200 and Panavia SA LUTING Plus), the glass ionomer (Fuji

I), and the resin reinforced glass ionomer cement (GC FujiCEM 2) to Cp Ti. The hypotheses included (1) there would be no significant difference among the μSBS values of the cements applied, and (2) thermal aging would not affect the μSBS of the cements utilized.

## Material and Methods

A total of 100 rectangular plates of Cp Ti (4×4×4 mm) (imes-icore GmbH, Eiterfeld,Germany) was fabricated using milling method and then were embedded in self-cured acrylic resin (Acropars, Marlic Co. Tehran, Iran). To make flat surfaces that were suitable for cement

bonding and SBS measurement, the Ti surfaces were serially polished with 400, 600, and 800 grit silicon carbide papers (Starcke, Hoffman Co, Germany) under cooling water flow. Then, the prepared samples were airborne-particle abraded with 250μm alumina (Bego, Bremen, Germany) at 0.4-MPa pressure for 10 seconds at 10-mm distance according to the manufacturer’s instructions. After that, the abraded samples were washed, cleaned with an ethanol solution and then were divided into 5 groups (n=20) according to the cement applied.

 Table 1 presented the dental cements that were used in this study. Panavia F2 (Kuraray Noritake Dental Inc, Kurashiki, Okayama, Japan) as a dual-cured self-etch resin cement were applied in group 1. At first, one drop of each bottle of A and B of ED primer mixed in a plastic well using a disposable brush and applied on the Cp Ti surfaces. Then, equal amount of paste A and B of the cement were mixed with a plastic spatula on a paper pad and then were pushed in the plastic molds with 3mm height and about 1 mm surface area which were held perpendicular over the Cp Ti substrates. The excess of the cements was removed with a sharp explorer from the periphery of the mold. The cement was polymerized for 40 s by a light curing device (Bluephase C8, Ivoclar Vivadent, Schaan, Liechtenstein) from above of each mold. The power density of light curing device was checked at first and after every 10 exposures. Two types of dual polymerizing self-adhesive resin cements; Rely X U200 (3M ESPE; St. Paul, MN.USA) and Panavia SA LUTING Plus (Kuraray Noritake Dental Inc, Kurashiki, Okayama, Japan) were used according to manufacturer’s instructions in group 2 and 3, respectively. A small amount of each cement inserted into the plastic molds and then was cured according to the protocols that previously were described.

**Table 1:**
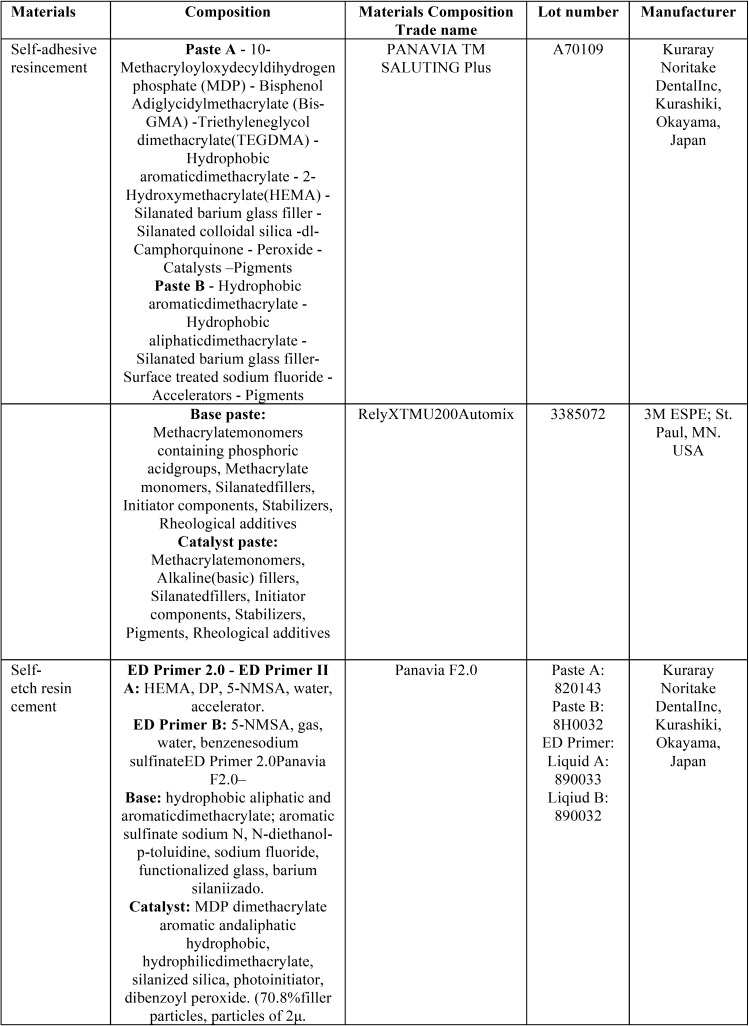
Luting cements were used in this study and composition information which provided by the manufacturers.

The self-cured Gl cement; Fuji I (GC, Tokyo, Japan) was applied in group 4 according to manufacturer’s instructions. In this manner the cement capsules were mixed in an amalgamator with 4000 rpm (SDS Kerr™ 4000 Amalgamator, Kerr Co, California, USA) for 10 s and then were injected within the tubes by a special gun. In the latest group (group 5), the RMGl cement; Automix FujiCEM 2 (GC, Tokyo, Japan) was used. A waiting period of about 5 min was necessary for obtaining the initial setting time for the both Gl-based groups.

The bonded specimens were stored in distilled water for 24 hours in the incubator with 37 °C temperature and 100% humidity.

Each group was divided into 2 equal subgroups according to the application of thermal aging (n=10). The half of the specimens was subjected to thermal aging by an automated thermal cycling machine (Nemo Co., Mashhad. Iran) with water temperatures between 5°C and 55°C for 5000 cycles and a 15-second dwell time.

-Debonding procedure

The plastic molds were separated by the sharp scalpel blade and carefully removed from the periphery of the polymerized cements. Then, they were mounted in a holding device within a universal testing machine (Santam, model STM-20, Tehran, Iran) to import the shear force to the adhesive interface until fracture was occurred. The bichisel was placed perpendicular to the metal-cement interface and the specimens were loaded at a speed of 1 mm/min. The SBS was calculated in megapascals (MPa) by dividing the load at the failure point (newtons) by the surface area of the metal-cement bonding (1.13 mm2).

-Fracture analysis

After the specimens were fractured and removed from the testing apparatus, the fracture sites were observed with the stereomicroscope (Dino lite Pro, Anmo Electronics Corp, Taiwan) at ×30 magnification to identify the type of bond failure. The fracture modes were classified to the (1) adhesive failure at the interface of the resin cement with Ti substrate; (2) the cohesive failure within the resin cement; and (3) the mixed failure mode, a combination of adhesive and cohesive failures.

-Statistical analysis

Data were analyzed using the SPSS statistical software (version 22.0, IBM, Chicago, IL, USA). To evaluate statistical significance among the study groups, a two-way analysis of variance (ANOVA) was conducted, followed by Tukey HSD test. All the statistical analysis was performed with a significance level set at 5%.

## Results

**Figure 1:**
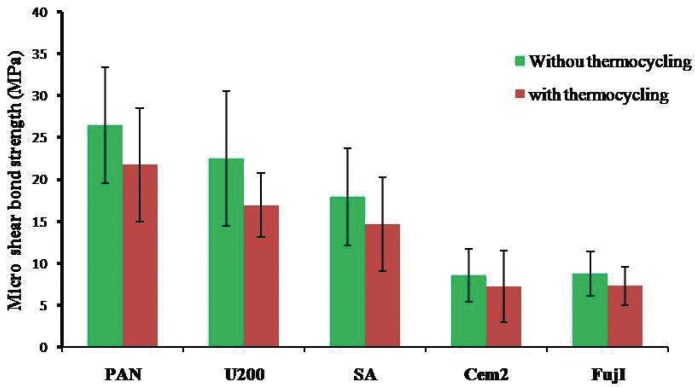
The mean µSBS values and standard deviations of all groups with and without aging condition. PAN: Panavia F.2, U200: Rely X U200, SA: Panavia SA LUTING Plus, Cem2: GC FujiCEM 2 and Fuji 1: GC Fuji I.

According to the outcome of Shapiro-Wilk analysis, all the experimental groups revealed the normal distribution (*P*>0.05). The two-way ANOVA showed significant effect of the luting cement type used and thermal aging on the μSBS values (respectively, *P*=<0.001 and *P*=0.003). However, no significant interaction between both variables was detected (*P*=0.62) ( Table 2). The mean, max and min of μSBS values and standard deviations of all groups with and without aging condition were presented in  Table 3 and also demonstrated in figure 1.

**Table 2:**
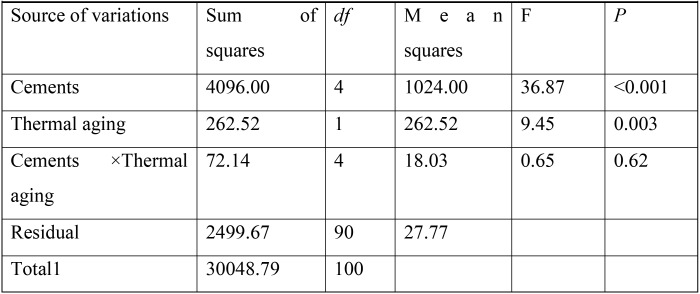
Two-way ANOVA analysis.

**Table 3:**
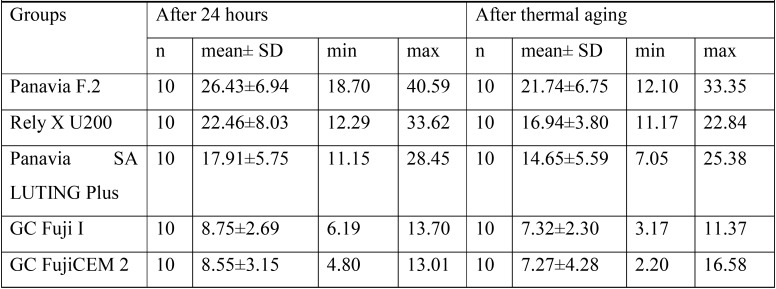
Mean, SD, min and max values of SBS (MPa) for all groups.

The total mean ±SD μSBS values ranged from 7.91 ±3.71 MPa (GC FujiCEM 2) to 24.09 ±7.08 MPa (Panavia F.2).

According the results from Tukey analysis, the μSBS value of Panavia F.2 was significantly higher than other groups (*P*<0.001) except Rely X U200 (*P*=0.07). In contrast, the GC Fuji I and GC FujiCEM 2 groups showed significantly lower μSBS values compared other three groups (*P*<0.001), but not significantly different from each other (*P*=1.0). Also, there was no significant differences between Rely X U200 and Panavia SA LUTING Plus (*P*=0.25).

Two-way analysis of variance revealed after thermal cycling, the mean μSBS in all study groups significantly decreased (*P*=0.003).

**Figure 2:**
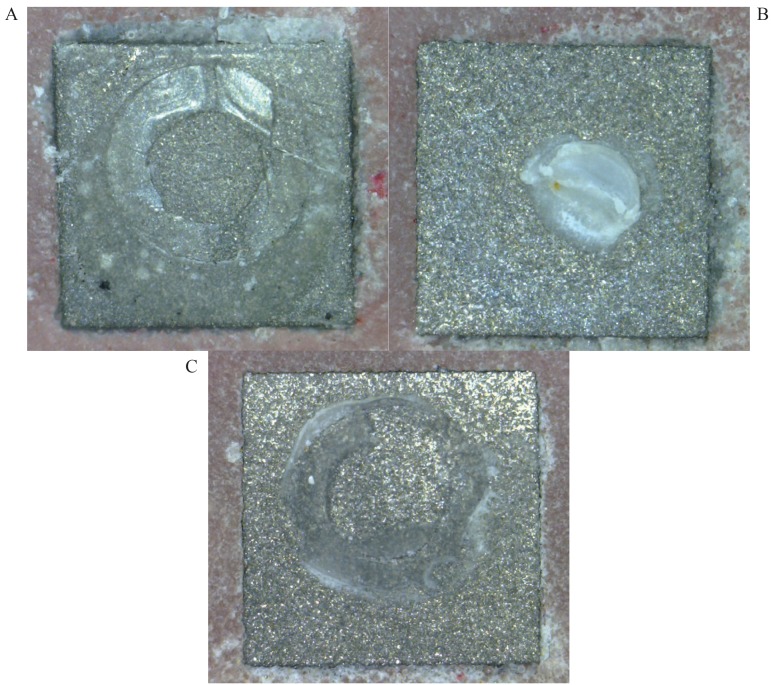
The three images which obtained under ×30 magnification of the stereomicroscope showed; A: the adhesive, B: cohesive and C: mixed failure modes.

The percents of failures modes are presented in  Table 4. The adhesive failure was reported as the main failure while a few cohesive and mixed failures were seen (Fig. 2).

**Table 4:**
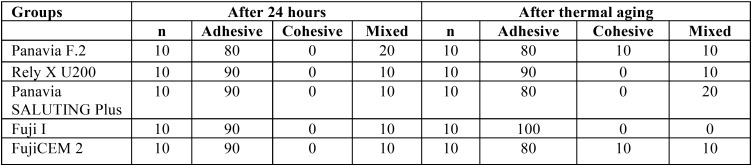
The percent of failure modes of study cements.

## Discussion

This study focused on the bonding effectiveness of different cement to Titanium (Ti) using the hybrid abutment concept. The hybrid or bi-component abutments have been developed to overcome the esthetic problem of metal abutments through joining the ceramic materials to Ti

platforms by the luting agants. Thus, the achievement of the strong adhesion between the ceramic components and Ti-based material for long term success of the prostheses restorations is required.

Since, shearing forces are the most predominant forces during mastication and chewing, the shear bond strength (SBS) was considered as the most commonly used methods to assess the metal–resin adhesion efficacy in the laboratory situation (17), as used in the current study.

In this study, grit-blasting on Ti surface with 250 μm Al2O3 particles is taken as a general treatment for all groups, based on previous studies reported the benefits of air-born particle abrasion in enhancing the bond and retention strength of different luting cements to metals (6,12,18). It seems the air-born abrasion improved the adhesion mechanisms between metal and resin cements through two mechanisms. In spite of increasing the surface roughness and micromechanical retention, the remaining of oxygen and aluminum particles on the sandblasted metal surface produce chemical affinity to functional monomers of resin materials (19).

The establishment of strong and durable adhesion between cement and Ti depends on the composition, properties and adhesive ability of the cement as well as the Ti surface properties. Therefore, in the current study, four different categories of luting cements were used. Regarding the obtained results from this study, the first null hypothesis that the type of cements would not affect the adhesion of the Cp Ti surfaces was rejected. The statistical analysis revealed there was a significant difference between study groups (*P*<0.001).

The results of the present study revealed the μSBS values of both Fuji I and FujiCEM 2 cements were significantly lower than resin cements (*P*<0.001). Fuji I is a brand of GI cement which provided advantages including low cost, relatively biocompatibility, fluoride release and simple manipulation (20). The FujiCEM 2 with the intermediate composition and properties between both of conventional Gl and resin cements (21), powered by F2 (Flex Fuse) technology which incorporates high-elastic cross-linking monomers with a modified filler-surface treatment to increase strength properties. In spite of these fact that both of GI-based cements contain carboxylic groups which could form chemical bonds with the oxide

layer on Ti surface (6), in the present research they could not enhance the μSBS. In contrast to this study that the lower μSBS of these cements (about 8 MPa) was consistent with the predominant adhesive failures, Fawzy *et al.* (6) reported higher SBS values for both Gl and RMGI cements (11-13 MPa) which associated with the predominant cohesive type of failure.

The authors concluded that the actual adhesive BS of these cements to Ti could be superior to the measured SBS and cohesive strength of these cements. In agreement with previous study, Weyhrauch *et al.* (22) reported greater retentive strength for Fuji CEM and Rely X Unicem (the self-adhesive resin cement) compared with Panavia F.2, when the e.max CAD crowns cemented on the Ti abutments. These controversies may be related to the complex nature of several bonded interfaces and substrates that can be affected the bonding performance in comparison with the present study that only one interface of cement- Ti evaluated. The weak adhesion of Rely X Luting 2 (6.31 ±1.30 MPa) which is a type of RMGl to Ti compared with other resin cements in Abi-Rached *et al.* study (3) was the same as the present study (8.55 ±3.15 MPa).

The adequate ranges of μTBS values for resisting against masticatory forces from in vitro studies was 15-20 MPa (23,24). Based on ISO 1047734 requirements (25), the minimum acceptable SBS value for the resin-based materials to different substrates is 5 MPa. In clinical situations, the resin-metal interface should be withstanded at least 10 MPa to achieve satisfactory results (26). In the present study, μSBS values of resin cements were above 10 MPa, while both Gl-based cements presented about 8 MPa which was lower than the acceptable level. Based aforementioned statements, the outcome of this study revealed the Fuji I and GC FujiCEM 2 could not provide the acceptable adhesion to Ti in clinical situations.

The results of the current study revealed the highest μSBS for the Panavia F.2 cement that was significantly different from the other study groups (*P*<0.001). A similar μSBS value was noted when Rely X U200 was applied (*P*=0.07). Panavia F.2 cement as the well-known

self-etch cement needs the preparation of the substrate with the primer before cement application. In contrast, the self-adhesive resin cements such as Rely X U200 needs no metal primers or silane coupling agents before cement application. Therefore, they minimize the required clinical steps and minimizing operator-related errors.

However, there are several studies evaluating the effect of different cements on Ti posts that cemented into the endodontically treated teeth (13-16), but few studies compared the traditional and resin cements that bonded to the Ti surfaces alone. The bonding of Ti posts to root dentin involves an adhesive joint with more than one interface. These interfaces are composed of different materials that do not necessarily bond equally well to luting agents (12). Therefore, the comparison of the result of those to the outcome of this study is not valid.

Ideally, to provide a reliable adhesion between the Ti surface and resin materials, a combination of micromechanical retention through sandblasting and chemical bonding is required (27). The comparable μSBS value of the Panavia F.2 and Rely X U200 in the current study may be related to the chemical affinity of phosphoric acid groups and MDP monomers of these cements with aluminum particles which trapped on Ti surface after sandblasting process (4,28). On the other hand, the layers of oxides that covered the pure Ti are produced during 9 to 10 s by contact with oxygen that guarantees the resin to metal adhesion through its reaction with functional monomers which derived from carboxylate (4-MET and 4-META) and phosphoric acid (MDP) (29). This interaction happened through hydrogen bridges by Bolger’s mechanism (30). In this mechanism, an electrostatic interaction between polymer acids or bases and hydroxyl groups of the metal surface would occur (31). However, Panavia F.2 and RelyX U200 showed SBS means without a statistical significant difference (*p* > 0.05), but did not show the same fracture failure mode. The more cohesive and mixed failures in Panavia F.2 compared with the most adhesive failures in Rely x U200 indicated a better clinical adhesive bond with Ti in Panavia F.2 that exceeded the cohesive strength of the cement.

Another factor that can be explicated the highest adhesion of Panavia F.2 and described by Schneider *et al.* (31) is wetting ability. The wetting ability of Panavia F.2 which related to the MDP and VBATDT monomers, resulting in the best contact area between the cement and Ti surface and improvement of adhesion.

In spite of the presence of MDP in Panavia SA LUTING Plus, but this cement could not present the comparable SBS with Panavia F.2. However, it produced the clinically acceptable adhesion to Ti (14-18 MPa). The lower SBS of the Panavia SA LUTING Plus than other two resin cement tested may be related to the HEMA monomers that incorporated into the cement formulation. HEMA is a hydrophilic monomer that is frequently added to the dentin adhesive or cements to improve adhesion with dentin. However, it presents several drawbacks. It was demonstrated the in vitro durability of commercial adhesives containing this monomer significantly decreased after water storage. The hydrophilic character of this monomer resulted retaining the water within the material, thereby weakening the mechanical strength of the adhesive or cement. Also, HEMA can be hydrolyzed in aqueous solutions. High water absorbency coupled with low polymerization reactivity of HEMA resulted in weak mechanical properties as well as poor bonding (32).

The thermocycling test as a screening tool could estimate the bonding durability in laboratory situations under standardized hydrothermal stresses. In this study, to compare the bond strength according to the storage time, half of the samples were submitted to 5000 cycles of thermocycling which were equivalent to approximately six months clinical service (33). Since the patients cannot tolerate the direct long-term contact of vital teeth with too cold or hot materials longer than 15s (34), water bath duration was set at 15 s. The obtained results presented thermocycling negatively influenced the adhesion between Ti and all tested cements (*P*=0.003). Thus, the second hypothesis was also rejected. Indeed, thermal cycling induced stresses the bond between Ti and resin cement due to differences in the coefficient of thermal expansion between Ti and the luting material used (5,35). However, the thermal stresses did not separate any of luting agent from Ti but they significantly decreased the μSBS of all cements (*P*=0.003). In contrast with the current investigation outcome, in Fonseca *et al.* (7) study, six month water storage had no adverse effect on the SBS of the Panavia F.2 cement to Cp Ti. The author attributed this outcome may be the results of insufficient storage time or the progression of the chemical reaction between the monomers and metal oxides. It was demonstrated the MDP monomer may be responsible for stability of some resin cements during storage or thermal aging (36). But this monomer could not decrease the deteriorating effect of thermal aging on the resin cements containing 10-MDP monomer in this experiment.

The failure analysis revealed all cements that tested in this study presented predominantly adhesive failures. The resin cement monomers contain many double bonded carbon units that could provide a high degree of matrix cross-linking and generates superior mechanical properties. In contrast, low cohesive strength of Gl resulted in the bulk of the material encountered a failure before debonding happened (3,6). However, in the current study, the adhesive failures were reported as a main failure in both Fuji I and Fuji CEM 2 groups.

The higher cohesive and mixed failure of Panavia F.2 compared with other study groups, especially after thermal aging in the present study indicates an acceptable and durable clinical adhesion with Ti. Indeed, the most wetting ability of Panavia F, as previously described, provided the high adhesion with Ti. But the putty consistency of this cement can be facilitated entrapping the bubbles during mixing two pastes of the cement which resulted in reduced cohesive strength; a fact that previously described by Schneider and co-workers (31).

Whereas, the other two resin cements; Rely X U200 and Panavia SA LUTING Plus with no mixing pastes minimize this phenomenon.

The results of this in vitro study suggest that use of resin cements combined with a mechanical retention, improved the bonding to Cp Ti. It seems the bonding efficacy of the self-adhesive resin cement seems to be related to the brand and materials. Since, Panavia F.2 and Rely X U200 cements yielded equivalent μSBS, they can be used for the bonding to Ti-base component in hybrid abutments. But in these situations, the utilizing of the Gl-based cements may not be sophisticated.

Finally, it should be noted some limitations of this study. The simulation of oral conditions and restorations in a laboratory was too difficult. The cubic forms of Ti which were used to provide basic information on cement adhesion did not simulate the true clinical situation of cement flow and distribution between the abutment and ceramic surfaces. In addition, the effect of unfavorable C factor (ratio of bonded to unbonded surface areas) of the cements and a relatively high volumetric shrinkage of the resin cement that happened in clinical situations did not simulate. In spite of thermal cycles, pH changes and dynamic fatigue loading may influence the durability of resin bonds, which were not evaluated in the present study.

Regarding to the mentioned limitation and prior to recommending the cement for clinical use, the long-term clinical studies should be conducted to affirm the efficacy of the cements that were used in the present study.

## Conclusions

Within the limitations of this in vitro study, the following conclusions were drawn:

1. The utilization of cements containing functional monomers including Panavia F.2 and Rely X U200 would be able to produce an effective adhesion to Ti.

2. The application of Gl (Fuji I) and RMGl (FujiCEM 2) did not suggest for bonding to Ti surfaces.

3. The bonding efficacy of the all types of cements, including resin-based and Gl- based deteriorated after thermal aging.
